# Crystal Structures and Physicochemical Properties of 3-Chloro-4-hydroxyphenylacetic Acid Salts with Amines

**DOI:** 10.3390/molecules28196965

**Published:** 2023-10-07

**Authors:** Remi Rolland Ngoma Tchibouanga, Ayesha Jacobs

**Affiliations:** Chemistry Department, Faculty of Applied Sciences, Cape Peninsula University of Technology, P.O. Box 1906, Bellville 7535, South Africa; olivianederolland@gmail.com

**Keywords:** 3-chloro-4-hydroxyphenylacetic acid, organic salts, aminopyridines, diethylamine, di-*N*-butylamine, crystal structures

## Abstract

3-chloro-4-hydroxyphenylacetic acid (CHPAA) is a fungal metabolite. It is a small molecule that is useful in crystal engineering studies due to the functional groups present. Six amines were selected to form salts with CHPAA. Linear derivatives included diethylamine (DEA) and di-*N*-butylamine (DBM). The aromatic compounds chosen were 2-aminopyridine (A2MP), 2-amino-4-methylpyridine (A24MP), 2-amino-6-methylpyridine (A26MP) and 4-dimethylaminopyridine (DMAP). The salts were characterised using single-crystal X-ray diffraction, thermal analysis, FTIR spectroscopy and Hirshfeld surface analysis. For all the crystal structures, N-H···O and C-H···Cl contacts were present. O-H···O contacts were found in all the crystal structures except for ($CHPAA^{2-})\left(2DEA^{+}\right)$, which was also the only structure that displayed a Cl···Cl contact. Furthermore, C-H···O contacts were found in all the crystal structures except for $(CHPAA^{-})(DBM^{+}).\;$The thermal stability trend showed that the DBM salt was more stable than the DEA salt. For the aromatic co-formers, the thermal stability trend showed the following: $\left(CHPAA^{-}\right)(DMAP^{+})\;$>$\;(CHPAA^{-})(A2MP^{+})>\left(2CHPAA^{-}\right)\left(2A26MP^{+}\right)>(CHPAA^{-})(A24MP^{+})$.

## 1. Introduction

**Scheme 1:**
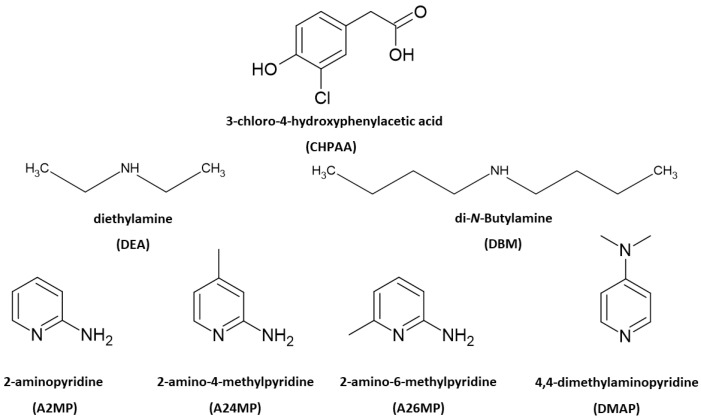
Chemical structures of CHPAA and the amines.

Multicomponent crystals are important in the pharmaceutical industry as the formation of new solid forms can improve the physicochemical properties of active pharmaceutical ingredients [1,2]. The choice of co-crystal formers can affect properties such as the solubility, hygroscopicity and compaction of the resultant pharmaceutical co-crystals and salts. Natural products, including small molecules derived from plants and fungi, also contribute to drug design. These metabolites are of interest due to their potential biological activity [3,4]. Crystal engineering principles of exploiting intermolecular interactions are utilised to obtain multicomponent crystals with suitable properties. These concepts are also relevant in the agrochemical industry [5]. One example is the improved solubility of the herbicide atrazine through co-crystallisation with fumaric acid [6]. Another herbicide 2,4-dichlorophenoxyacetic acid, formed salts with imidazole, 2-aminopyridine and 3-aminopyridine and co-crystals with isonicotinamide and pyrazinamide. All five multicomponent crystals showed enhanced solubility compared to the 2,4-dichlorophenoxyacetic acid alone [7]. Intermolecular interactions such as hydrogen bonding, π–π stacking and halogen bonding are typically employed in the formation of new solid forms. Strong hydrogen bonds include O-H···O and N-H···O. Examples of weak hydrogen bonds are C-H···O, C-H···π, O-H···π and C-H···Cl [8]. Crystal engineering employs supramolecular synthons whereby functional groups present in molecules form complementary units [9]. Etter’s hydrogen bond rules provide insights into the organisation of crystal structures with a focus on the best proton donor pairing with the best acceptor group [10]. The interaction of identical functional groups gives rise to homosynthons, whereas heterosynthons result when different functional groups interact. Notable heterosynthons are the robust carboxylic acid pyridine and carboxylic acid 2-aminopyridinium building blocks [11,12]. Amines and carboxylic acids also form the useful N-H···O heterosynthon [13]. In this study, we continue our focus on the crystal landscape of the fungal metabolite 3-chloro-4-hydroxyphenylacetic acid (CHPAA). CHPAA has previously been isolated from natural sources [14] and has also been used in the development of a screening library for applications in medicinal chemistry [15]. A library of compounds based on the skeletal framework of 3-chloro-4-hydroxyphenylacetamide and CHPAA has also been developed for potential use in pharmaceutical and agrochemical screening [16]. CHPAA is useful in crystal engineering studies as it possesses carboxylic acid and hydroxyl functional groups. Furthermore, CHPAA also contains the chlorine atom in the meta position, which can be involved in hydrogen and/or halogen bonds. We have previously reported co-crystals of CHPAA with nicotinamide, isonicotinamide, phenazine and 4,4′-bipyridine [17]. These are the only crystal structures of CHPAA in the CSD version 5.44, June 2023 update [18]. In the current study, a series of linear amines and aminopyridine derivatives were selected to co-crystallise with CHPAA. These included diethylamine (DEA), di-*N*-butylamine (DBM), 2-aminopyridine (A2MP), 2-amino-4-methylpyridine (A24MP), 2-amino-6-methylpyridine (A26MP) and 4,4-dimethylaminopyridine (DMAP). These are depicted in Scheme 1. The p*K*_a_ [19] of CHPAA is 3.06 and the p*K*_a_ values of the amines are DEA (10.58), DBM (10.75), A2MP (6.84), A24MP (7.62), A26MP (7.60) DMAP (8.78). Thus, the ∆p*K*_a_ values ranged from 3.78 to 7.69. Salt formation is expected when ∆p*K*_a_ > 4. If <−1 ∆p*K*_a_ < 4, then either salt or co-crystal can form [20]. In this study, proton transfer occurred between the CHPAA and the respective bases forming salts. This work aimed to prepare multicomponent crystals of CHPAA with the abovementioned amines and to study their crystal structures and thermal stability. Fourier transform infrared (FTIR) spectroscopy was used to confirm the formation of the new solid forms. Furthermore, alternate methods of preparation of the salts were also explored, for example, liquid-assisted grinding and slurry conversion, and the products were analysed using powder X-ray diffraction. Hirshfeld surface analysis was used to compare the intermolecular interactions in the salts, and the resulting fingerprint plots were interpreted.

## 2. Results and Discussion

### 2.1. Crystal Structures

**Figure 1:**
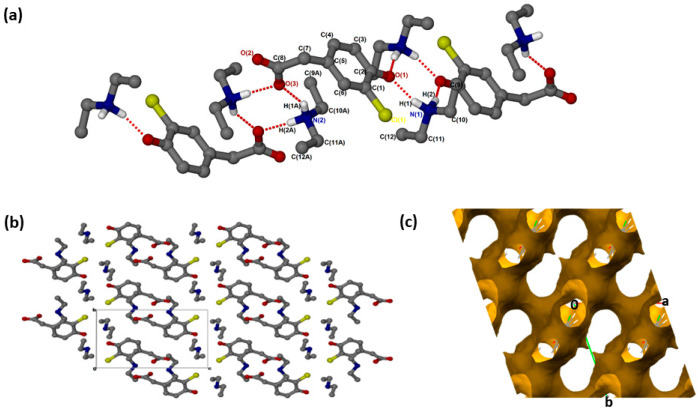
(**a**) Hydrogen bonding in ($CHPAA^{2-})\left(2DEA^{+}\right)$, (**b**) Packing diagram along [100] and (**c**) $DEA^{+}$ removed to show voids along [001].

($CHPAA^{2-})\left(2DEA^{+}\right)\;$crystallised in the triclinic space group *PĪ*, Z = 2 (Figure 1a). The crystal data is summarised in Supplementary Materials: Table S1. In addition to proton transfer from the carboxylic acid to the nitrogen of one of the DEA bases, the hydroxyl hydrogen of the CHPAA molecule was transferred to the nitrogen of the second DEA base. ($CHPAA^{2-})\left(2DEA^{+}\right)$ presents two major N-H···O intermolecular interactions, N1-H1···O1 and N2-H1A···O3, generating two rings which can be described in graph set notation as $R_{4}^{2}\left(8\right)$ rings [21]. There are C-H···O contacts with *d*(C11···O2) of 3.4706(18) Å as well as contacts to the chlorine atom; C-H···Cl (d(C11···Cl1) = 3.821(2) Å) and N-H···Cl (d(N1···Cl1) = 3.4818(16) Å). The hydrogen bond metrics are summarised in Table S2. The chlorine atoms of adjacent CHPAA^2−^ anions point directly towards each other, forming a type I halogen bond with *d*(Cl···Cl) = 3.517(2) Å and an angle of 174° (Figure 1b). The Cl···Cl distance in ($CHPAA^{2-})\left(2DEA^{+}\right)$ is close to twice the value of the van der Waals radius of Cl (1.76 Å) [22]. Two models have been proposed for Cl···Cl interactions: the Nyburg model [23,24] and William’s model [25]. Subsequently, the nature of Cl···Cl interactions has been the subject of much debate [26,27,28]. The Cl···Cl distance in ($CHPAA^{2-})\left(2DEA^{+}\right)$ is also similar to those found in substituted 2-chloroquinoline derivatives [29]. The DEA^+^ cations occupy channels (Figure 1c) within the crystal structure; the voids were calculated using Mercury, with a probe radius of 1.2 Å, and found to constitute 62.6% of the unit cell.

**Figure 2:**
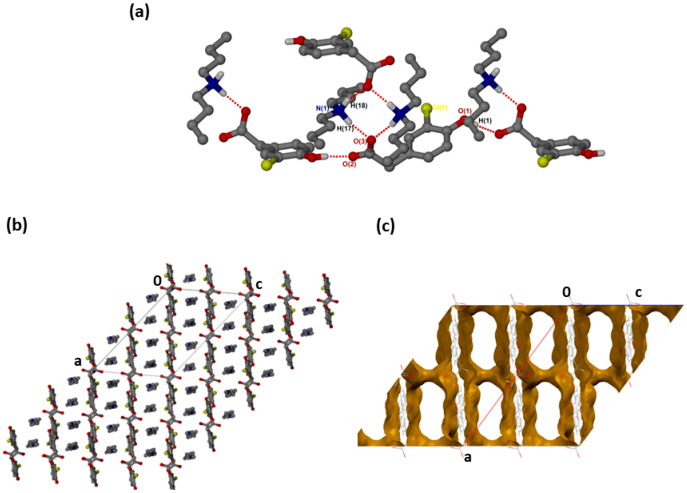
(**a**) Hydrogen bonding in (CHPAA^−^)(DBM^+^), (**b**) Packing diagram along [010] and (**c**) DBM^+^ removed to show voids along [010].

$\left(CHPAA^{-})(DBM^{+}\right)$ was successfully solved in the monoclinic space group *C2/c* with Z = 8 (Figure 2a). Both N-H groups are involved in hydrogen bonding to a carboxylate forming $R_{4}^{2}\left(8\right)$ rings. The CHPAA^−^ hydroxyl group also forms O-H···O hydrogen bonds to neighbouring carboxylate oxygens, generating a chain with a graph set notation of$\;C_{1}^{1}\left(9\right)$. The closest contacts to the chlorine atom are from the alkyl chain of $DBM^{+}\;$with d(C10···Cl) of 3.7662(15) Å and d(C16···Cl) of 3.6809(17) Å. CHPAA^−^ is oriented to form chains, allowing the DBM^+^ cations to accommodate in channels (Figure 2b). The void space occupied by DBM^+^ cations is 59.7% of the unit cell (Mercury, probe radius 1.2 Å).

**Figure 3:**
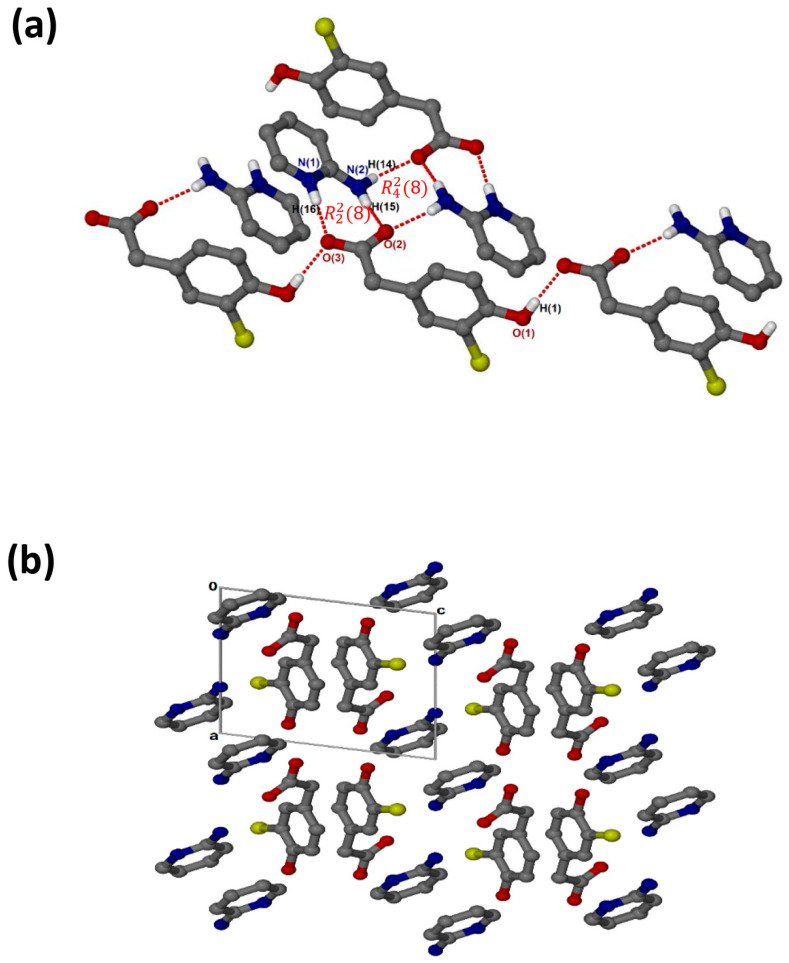
(**a**) Hydrogen bonding in $\left(CHPAA^{-})(A2MP^{+}\right)$ and (**b**) Packing diagram along [010].

$\left(CHPAA^{-})(A2MP^{+}\right)$ crystallised in the triclinic space group *PĪ*, Z = 2 (Figure 3a). There are N-H···O hydrogen bonds, as well as O-H···O linkages between the hydroxyl group and neighbouring carboxylate oxygens. The $A2MP^{+}$ cations and the carboxylate oxygens form $R_{2}^{2}\left(8\right)$ aminopyridinium carboxylate heterosynthon motifs with the hydrogen bonding extended to include the pyridinium nitrogen, which forms additional $R_{4}^{2}\left(8\right)$ motifs. There are weak contacts C12-H12···π (3.452(1) Å), C6-H6···π (3.857(1) Å) and C10-H10···Cl (3.7854(17) Å). As seen for ($CHPAA^{2-})\left(2DEA^{+}\right)$, C-H···O contacts (d(C10···O1) = 3.367(2) Å and (d(C9···O3) = 3.461(2) Å) are also present. In the packing diagram below (Figure 3b), the cations and anions are arranged in alternate columns with the packing stabilised by π–π stacking between CHPAA^−^ anions (3.639(1) Å) and between 2*AMP^+^* cations (3.835(1) Å).

**Figure 4:**
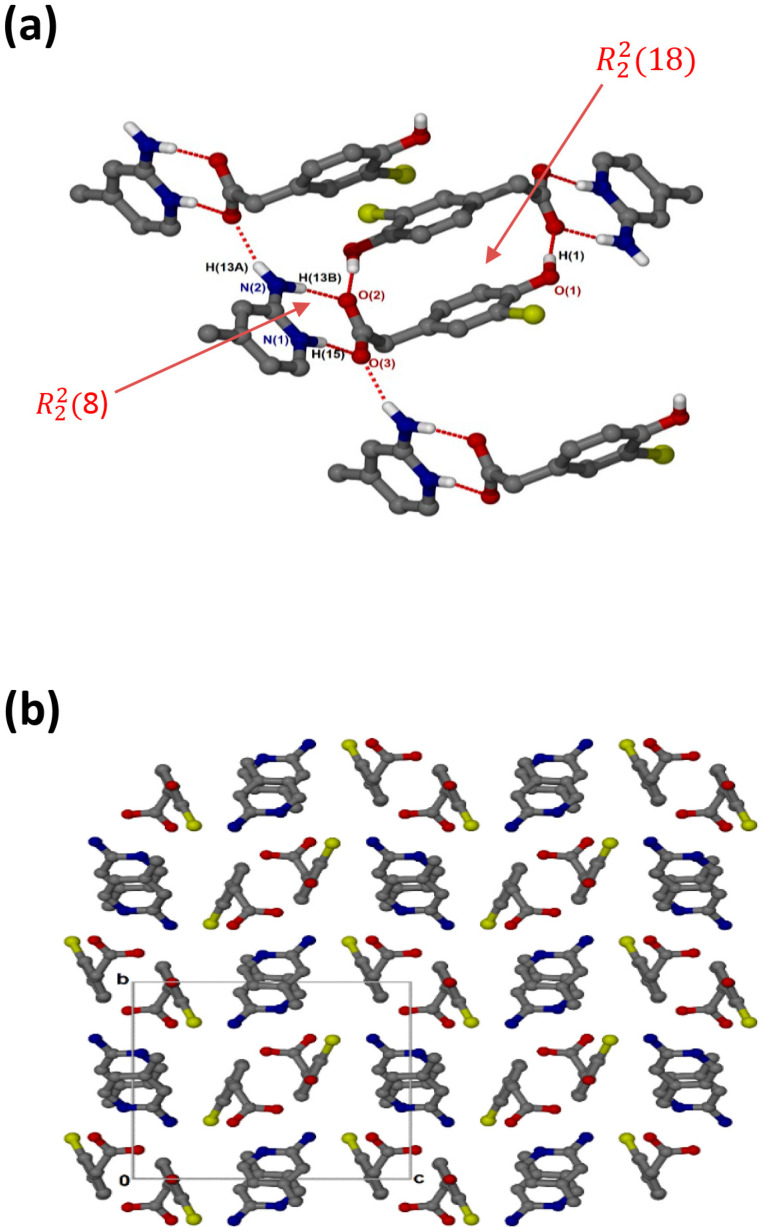
(**a**) Hydrogen bonding in $\left(CHPAA^{-}\right)\left(A24MP^{+}\right)$ and (**b**) Packing diagram along [100].

$\left(CHPAA^{-}\right)\left(A24MP^{+}\right)$ crystallises in the monoclinic space group *P2_1_/c* and its asymmetric unit contains one $CHPAA^{-}$ anion and one $A24MP^{+}$ cation (Z = 4). The 1:1 salt of ($CHPAA^{-})\left(A24MP^{+}\right)$, shows that the carboxylate and aminopyridinium interactions also form the $R_{2}^{2}\left(8\right)\;$supramolecular heterosynthon. However, in this structure (Figure 4a), the acid anions dimerise to form the $R_{2}^{2}\left(18\right)$ homosynthon involving the hydroxyl group and carboxylate oxygen (O-H···O interaction). Furthermore, the hydrogen *anti*-oriented (H13A) on the primary amine forms an additional hydrogen bond, allowing the structure to generate an extended network system described as chains of$\;C_{2}^{2}\left(8\right)$ and $C_{2}^{2}\left(6\right)$ motifs via N-H···O interactions. The chlorine atom also interacts with one of the carbons of the cation, forming a weak C3-H3···Cl1 interaction. This generated the closest contact of 3.5088(16) Å for d(C3···Cl1) with the angle of 99.9°. There are also C-H···O contacts with C14-H14···O1 (3.405(2) Å) and C7-H7A···O2 (3.527(2) Å). Columns of cations and anions (Figure 4b) are arranged in an alternating fashion along [100]. The packing is further stabilised by π–π stacking, including acid–acid (3.602(1) Å) and cation–cation (3.746(1) Å).

**Figure 5:**
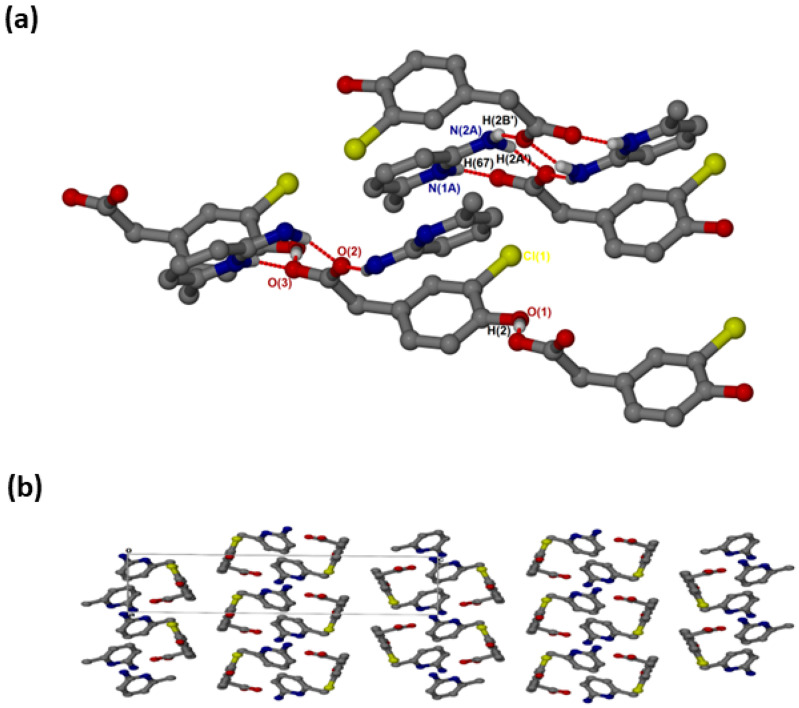
(**a**) Hydrogen bonding in $\left(2CHPAA^{-}\right)\left(2A26MP^{+}\right)$ and (**b**) Packing diagram along [100].

$\left(2CHPAA^{-}\right)\left(2A26MP^{+}\right)$ crystallised in the triclinic space group *PĪ* and its asymmetric unit contains two $CHPAA^{-}$ anions and two $A26MP^{+}$ cations (Z = 4). In the 2:2 salt of $\left(2CHPAA^{-}\right)\left(2A26MP^{+}\right)$, the anions$\;CHPAA^{-}$ interact with $A26MP^{+}$ via supramolecular heterosynthons (Figure 5a) consisting of $R_{2}^{2}\left(8\right)\;$and $R_{4}^{2}\left(8\right)\;$rings. The hydroxyl group is also involved in O-H···O interactions with the carboxylate oxygen of neighbouring $CHPAA^{-}$ anions forming $C_{1}^{1}\left(9\right)$ chains. A weak C9A-H9A’···Cl intermolecular interaction (3.823(4) Å) was also observed. The packing diagram (Figure 5b) shows columns of cations located between two columns of anions parallel to the *a*-axis. The π-π stacking interactions between $A26MP^{+}\;$cations (4.433(1) Å) are much weaker in this structure compared to $\left(CHPAA^{-})(A2MP^{+}\right)$ and $\left(CHPAA^{-}\right)\left(A24MP^{+}\right)$. C-H···π contacts between $A26MP^{+}$ cations and the aromatic ring of the $CHPAA^{-}\;$anion are also present with C13-H13···π (3.420(1) Å, 145°) and C13A-H13A···π (3.440(1) Å, 145°). There are also C-H···O contacts: C9A-H9A’···O1 (3.507(4)) Å and C9-H9A···O1A (3.523(5) Å).

**Figure 6:**
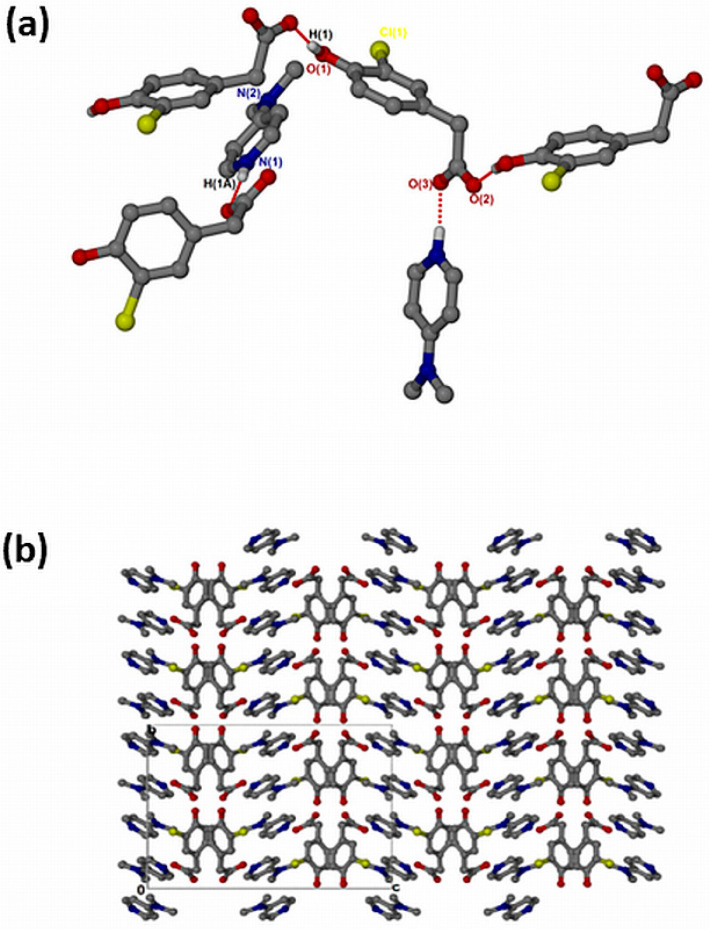
(**a**) Hydrogen bonding in $(CHPAA^{-})\left(DMAP^{+}\right)$ and (**b**) Packing diagram along [010].

The salt of 4-dimethylaminopyridinium-3-chloro-4-hydroxyphenylacetate, $(CHPAA^{-})\left(DMAP^{+}\right)$, crystallised in the orthorhombic space group *Pbca* and its asymmetric unit contains one $CHPAA^{-}$ anion and one $DMAP^{+}$ cation (Z = 8). The 1:1 salt of$\;\left(CHPAA^{-}\right)\left(DMAP^{+}\right)$ forms intermolecular interactions via N-H···O. The neighbouring hydroxyl group also interacts with the free O2 of another carboxylate forming extended columns with a $C_{1}^{1}\left(9\right)$ motif via O-H···O interactions (Figure 6a). The structure does not contain any hydrogen-bonded ring motifs as was seen in the previous structures due to the absence of the NH_2_ group in DMAP. There are π–π stacking interactions between adjacent $DMAP^{+}$ cations (3.807(1) Å). The structure also contains C-H···Cl contacts ranging from 3.474(3) Å for C13-H13···Cl1 to 3.846(4) for C15-H15B···Cl1 and C-H···O contacts range from 3.281(3) Å for C10-H10···O1 to 3.386(3) for C7-H7B···O1. The packing is shown in Figure 6b, highlighting a columnar arrangement parallel to the *b*-axis.

In summary, ($CHPAA^{2-})\left(2DEA^{+}\right)$ is the only structure that presents with a Cl···Cl contact and an N-H···Cl contact. All the structures exhibited C-H···Cl contacts with some longer than the van der Waals cut off, which is not unusual for weak interactions [5]. The findings are consistent with those found in a previous study involving CHPAA with nicotinamide, isonicotinamide, phenazine and 4,4′-bipyridine where none displayed Cl···Cl contacts and three of the structures contained C-H···Cl contacts. Crystal structures of the related compound, 2,4-dichlorophenoxyacetic acid with imidazole, 2-aminopyridine, 3-aminopyridine, isonicotinamide and pyrazinamide displayed similar C-H···Cl contacts [7]. Roy et al. studied salts of dicyclohexylamine with several substituted phenylacetic acids, including 2-, 3- and 4-chlorophenylacetic acid. In their supporting information, the authors reported the hydrogen bond metrics and tabulated N-H···O and C-H···O contacts for the 2-chlorophenylacetate and the 3-chlorophenylacetate salts. For the 4-chlorophenylacetate salt, the authors noted N-H···O as well as a C-H···Cl and a Cl···Cl contact [30]. N-H···O hydrogen bonds dominate for all the CHPAA structures and O-H···O for all except in the case of ($CHPAA^{2-})\left(2DEA^{+}\right)$. All the structures except for (CHPAA^−^)(DBM^+^) displayed C-H···O interactions. The O-H···O and N-H···O hydrogen bonds gave contact distances with average values for d(H···O) = 1.79 Å, d(H···N) = 1.89 Å, d(O···O) = 2.62 Å and d(N···O) = 2.78 Å. These were followed by the C-H···O and C-H···Cl contacts which gave average values of d(H···O) = 2.55 Å, d(H··· Cl) = 3.01 Å, d(C···O) = 3.44 Å and d(C···Cl) = 3.72 Å.

### 2.2. Hirshfeld Analysis

**Figure 7:**
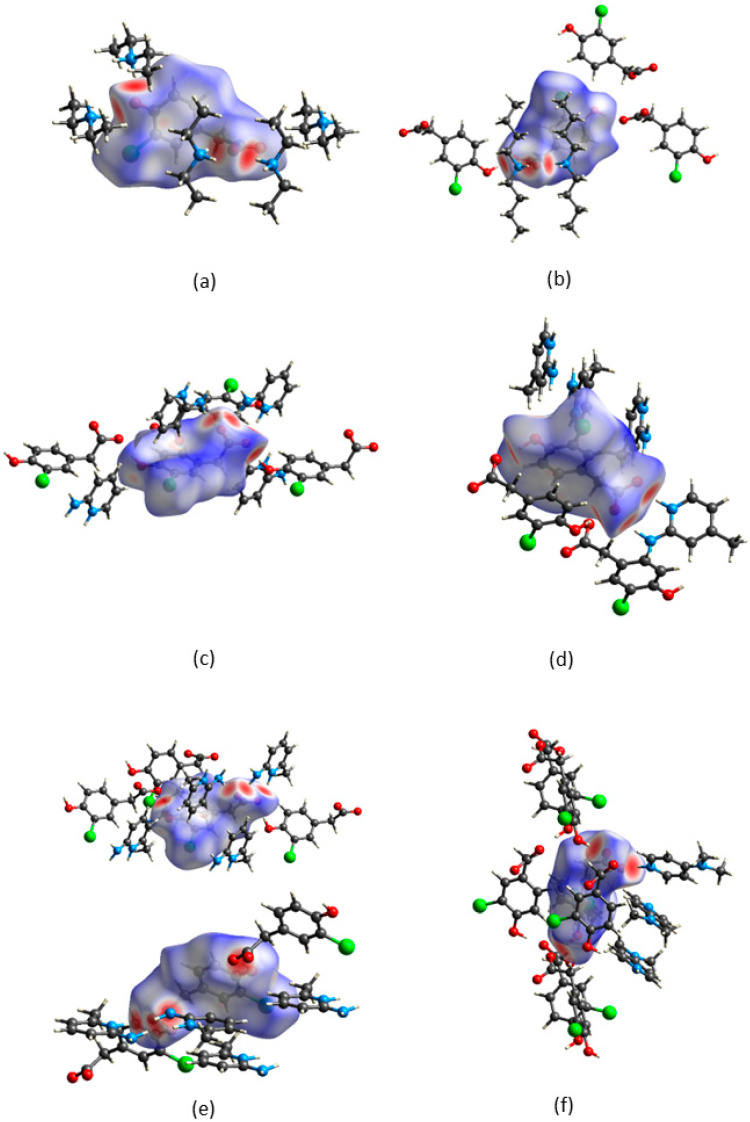
Hirshfeld surfaces mapped over d_norm_ for (**a**) (CHPAA^2−^)(2DEA^+^), (**b**) (CHPAA^−^)(DBM^+^), (**c**) (CHPAA^−^)(A2MP^+^), (**d**) (CHPAA^−^)(A24MP^+^), (**e**) (2CHPAA^−^)(2A26MP^+^) and (**f**) (CHPAA^−^)(DMAP^+^).

**Figure 8:**
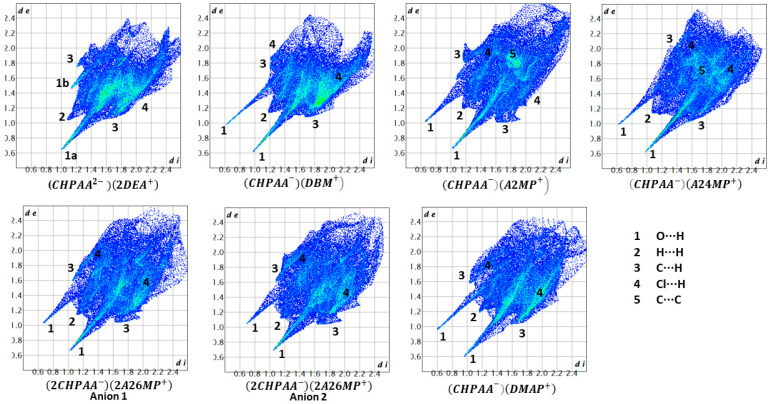
Fingerprint plots for the different salts.

**Figure 9:**
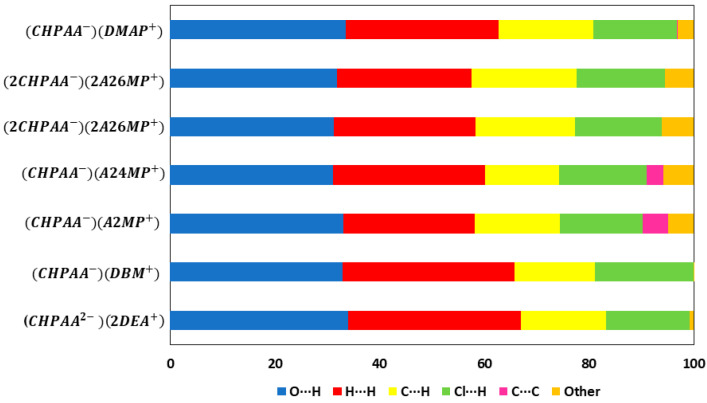
Graph showing the percentage contributions for various contacts in the different salt structures.

CrystalExplorer [31] was employed to generate Hirshfeld surfaces mapped with d_norm_ of the 3-chloro-4-hydroxyphenylacetate anion. Close contacts shorter than the sum of the van der Waals radii are highlighted in red, and the molecules involved are also represented (Figure 7). The bright red regions are associated with N-H···O and O-H···O contacts, whereas the lighter red areas are due to C-H···O contacts. The 2D fingerprint plots are shown in Figure 8. The main features of the packing for all the structures involve O···H, H···H, C···H and Cl···H contacts. The highest percentages were found for O···H (31.1–33.9%) and H···H (25.2–33%). The dominance of O···H contacts is consistent with the presence of N-H···O, O-H···O and C-H···O hydrogen bonds in the structures. Although there are no O-H···O hydrogen bonds in ($CHPAA^{2-})\left(2DEA^{+}\right)$ due to the deprotonation of both the carboxylic acid and the free hydroxyl, there are several N-H···O and C-H···O hydrogen bonds in this structure. In the 2D fingerprint plot for ($CHPAA^{2-})\left(2DEA^{+}\right)$, the long spike labelled **1a** is due to contacts between N-H of the DEA^+^ and the oxygen of CHPAA^2−^. Contacts between C-H of CHPAA^2−^ and oxygen outside the Hirshfeld surface result in the short spike labelled **1b**. For all the other structures, the two spikes labelled **1** have similar lengths due to the presence of the free hydroxyl group resulting in reciprocal O···H contacts. All the structures displayed significant C···H (14.1–20%) and Cl···H (15.8–18.8%) contacts. There are prominent wings on the fingerprint plots for$\;(CHPAA^{-})(A2MP^{+})\;$and $\left(2CHPAA^{-}\right)\left(2A26MP^{+}\right)$ due to C-H···π interactions. C···C contacts were observed for $\left(CHPAA^{-})(A2MP^{+}\right)$: 4.8% and $\left(CHPAA^{-})(A24MP^{+}\right)$: 3.2% which is attributed to the π···π stacking between CHPAA^−^ anions in these structures. Only ($CHPAA^{2-})\left(2DEA^{+}\right)$ displayed Cl···Cl contacts, although it was a very small percentage (0.8%). This is consistent with CHPAA containing one chlorine atom. A quantitative summary of the contributions is given in Table S3 and Figure 9.

### 2.3. Thermal Analysis

Differential scanning calorimetry (DSC) results are presented for all the salts, and thermogravimetric analysis (TGA) was completed for ($CHPAA^{2-})\left(2DEA^{+}\right)\;$and $(CHPAA^{-})(DBM^{+}).\;$The thermal analysis curves are given in Figure S1, and the results are in Tables S4 and S5. Two mass loss steps were observed for ($CHPAA^{2-})\left(2DEA^{+}\right)$. The first mass loss of 10.5% observed at 315 K is due to the loss of ½DEA, and the second mass loss at 396 K for the remaining DEA overlaps with the decomposition of CHPAA. The DSC curve of ($CHPAA^{2-})\left(2DEA^{+}\right)$ shows two endothermic peaks. The first peak at 316 K corresponds to the partial decomposition of DEA and is in agreement with the TGA result. The second peak at 391 K is due to the melt of CHPAA and the loss of the remaining DEA. For $\left(CHPAA^{-})(DBM^{+}\right)$, a continuous mass loss was observed at 400 K due to the loss of DBM and the melt of the CHPAA. The higher temperature at which DBM was released correlates to its higher normal boiling point (433 K) compared to DEA (normal boiling point 329 K). The remaining salts involving the aromatic amines had melting points higher than the starting materials. $\left(CHPAA^{-})(A2MP^{+}\right)$ melt occurred at $T_{onset}$ = 423.3 K (CHPAA: $T_{onset}$ = 378.3 K and A2MP: $T_{onset}$ = 331.4 K). For $\left(CHPAA^{-})(A24MP^{+}\right)$ the melting point is $T_{onset}$ = 410.7 K (A24MP: $T_{onset}$ = 365.8 K). In the case of $\left(2CHPAA^{-})(2A26MP^{+}\right)$, $T_{onset}$ = 414.8 K (A26MP: $T_{onset}$ = 306.7 K). $\left(CHPAA^{-})(DMAP^{+}\right)$ showed a melting peak at $T_{onset}$ = 427.6 K (DMAP^+^: *T_onset_* = 383.9 K). Thus, salt formation with the aromatic amines resulted in increased thermal stability with the highest melting point observed for $\left(CHPAA^{-})(DMAP^{+}\right)$.

### 2.4. Infrared Spectroscopy 

FTIR spectroscopy is often used in screening new solid forms to distinguish between co-crystal and salt formation [32,33]. It is particularly useful in the study of carboxylic acids as after salt formation, the characteristic -OH stretching band of the -COOH group will shift to form COO^−^ stretching bands. The CHPAA spectrum displayed a stretching peak at around 3431 cm^−1^ that is assigned to the carboxylic acid-free OH. This peak is absent or shifted in all the new solid forms, which is indicative of salt formation. CHPAA also contains one major stretching peak at 1694 cm^−1^ assigned to C=O. This peak was absent in all the new solid forms. The peaks at 1545–1571 cm^−1^ in the IR spectra of salts with aromatic amines were assigned to the COO^−^ group. In the salts formed from linear amines, the peak due to the carboxylate appeared at approximately 1640 cm^−1^. Furthermore, the band due to the NH stretch in A2MP (3444 cm^−1^), A24MP (3429 cm^−1^) and A26MP (3461 cm^−1^) either disappeared or diminished in the IR spectra of the resulting salts. This can be attributed to the −NH_2_ group’s involvement in hydrogen bonding in the A2MP, A24MP and A26MP salts. The FTIR spectra are given in Figures S2 and S3.

### 2.5. Grinding and Slurry Experiments

PXRD analyses of all compounds obtained from the grinding and slurry experiments were performed to determine whether the salts obtained by the slow evaporation technique can also be prepared using other techniques. The calculated PXRD patterns obtained from LAZYPULVERIX [34] were compared to the PXRD of the CHPAA starting material, as well as those obtained from the various preparation methods (Figure S4). The calculated PXRD pattern of ($CHPAA^{2-})\left(2DEA^{+}\right)$ was a good match with the slurry PXRD pattern. The PXRD pattern of the ground material still contained peaks found in the starting material, indicating that the reaction was incomplete. For CHPAA and DBM, the PXRD patterns of the slurry and the ground product were a perfect match, with additional peaks present compared to that of the calculated pattern, indicating an incomplete reaction. All PXRD patterns of the resulting salts of CHPAA with A2MP, A24MP and A26MP were similar to those of the calculated PXRD patterns, indicating that the slurry and ground preparations were successful in forming the required salts. In the case of CHPAA and DMAP, the grinding and slurry PXRD patterns were similar but contained additional peaks not found in the calculated PXRD pattern, which can be ascribed to a partial reaction.

## 3. Materials and Methods

All chemicals were purchased from Sigma Aldrich (Schnelldorf, Germany) and were used as received.

### 3.1. Crystallisation

($CHPAA^{2-})\left(2DEA^{+}\right)$ and $\left(CHPAA^{-})(DBM^{+}\right)$: CHPAA (30 mg, 0.161 mmol) was dissolved in 3 mL of the respective amine and a few drops of ethanol with heating to form solutions. The solutions were sealed and kept at room temperature to evaporate. ($CHPAA^{2-})\left(2DEA^{+}\right)$ formed yellow-green crystals and $\left(CHPAA^{-})(DBM^{+}\right)$ presented as dark yellow crystals after a few weeks.

$\left(CHPAA^{-})(A2MP^{+}\right)$: CHPAA (30 mg, 0.161 mmol) and A2MP (15.13 mg, 0.161 mmol) were dissolved in ethanol and a few drops of chloroform to form dilute solutions with heating. The solutions were sealed and kept at room temperature to evaporate. After 1 week, dark yellow crystals were obtained.

$\left(CHPAA^{-})(A24MP^{+}\right)$, $\left(2CHPAA^{-})(2A26MP^{+}\right)$: CHPAA (30 mg, 0.161 mmol) and the respective amine (17.385 mg, 0.161 mmol) were dissolved in excess ethanol with heating to form solutions. Slow evaporation at room temperature resulted in yellow-green crystals for $\left(CHPAA^{-})(A24MP^{+}\right)$ and colourless crystals for $\left(2CHPAA^{-})(2A26MP^{+}\right)$.

$\left(CHPAA^{-})(DMAP^{+}\right)$: CHPAA (30 mg, 0.161 mmol) and DMAP (19.633 mg, 0.161 mmol) were dissolved in excess ethylacetate with heating. Yellow-green crystals were obtained after one week of slow evaporation at room temperature. 

### 3.2. Grinding and Slurry Experiments

($CHPAA^{2-})\left(2DEA^{+}\right)$ and $\left(CHPAA^{-})(DBM^{+}\right)$: CHPAA and the respective amine were mixed with the addition of a few drops of ethanol. The mixtures were manually ground for 15 min using a mortar and pestle. For the slurry experiments, 30 mg of CHPAA and an excess of amine, together with a few drops of ethanol, were heated until a solution was obtained. The solution was removed from the heat and stirred continuously until a powder formed. The resulting powder was filtered and left to dry at ambient temperature.

$\left(CHPAA^{-})(A2MP^{+}\right)$, $\left(CHPAA^{-})(A24MP^{+}\right)$ and $\left(CHPAA^{-})(DMAP^{+}\right)$: The grinding and slurry experiments were performed in a similar manner to the above using equimolar mixtures of the acid and the amine.

### 3.3. Crystal Structure Analysis

Suitable crystals were selected for single crystal X-ray diffraction analysis at 173 (2) K. Diffraction data for all compounds were collected on a Bruker APEX II diffractometer (Bruker, Karlsruhe, Germany, with a graphite monochromated MoKα (λ = 0.71073 Å at 173 K). SADABS [35] was used to correct the intensities collected for absorption. The structures were solved by direct methods using SHELX-97 [36] and refined using full-matrix least squares methods in SHELXL. The graphical interface used was X-SEED [37]. All hydrogen atoms were placed geometrically with a riding model for their isotropic temperature factors except for those involved in hydrogen bonding, which were found in the electron density map and refined isotropically.

### 3.4. Powder X-ray Diffraction

A D2 PHASER Bruker diffractometer with Cu-Kα radiation (1.54184 Å) was used for PXRD. The voltage tube and current were at 30 kV and 10 mA max, respectively, with a scintillation counter 1-dim LYNXEYE detector. The scanning process of each sample was between 4–50° 2θ.

### 3.5. Infrared Spectroscopy 

Spectra were obtained from the universal attenuated total reflectance (UTAR) infrared spectrometer Perkin Elmer spectrum two. Sample spectra were measured over the range of 4000–400 cm^−1^.

### 3.6. Thermal Analysis

DSC analyses were performed on a Perkin-Elmer 6 system with a purge of nitrogen at 20 mL min^−1^. These analyses were conducted from 303–573 K with a heating rate of 10 K min^−1^.

Samples of 2–5 mg were removed from the mother liquor, dried with filter paper, then crushed to a fine powder and placed in a vented pan for the DSC analysis.

## 4. Conclusions

Salts of CHPAA with selected linear and aromatic amines were successfully prepared. The formation of the new solid forms was validated using DSC analysis, and in the case of the aromatic amines, the resulting salts demonstrated enhanced thermal stability compared to the starting materials. FTIR spectroscopy confirmed salt formation. The salt structures of CHPAA, except for ($CHPAA^{2-})\left(2DEA^{+}\right)$ demonstrated N-H···O and O-H···O hydrogen bonds as the major interactions. In the case of ($CHPAA^{2-})\left(2DEA^{+}\right)$, N-H···O contacts dominated. All the structures exhibited C-H···Cl contacts, which shows the significance of this interaction in stabilizing the structures. C-H···O contacts were found in all the structures except for (CHPAA^−^)(DBM^+^). The Cl···Cl halogen bond was only found in ($CHPAA^{2-})\left(2DEA^{+}\right)$, which was also the only structure that presented with an N-H···Cl contact. CHPAA utilised all its hydrogen bond donor and acceptor sites in the formation of the salts. 

## Data Availability

The supplementary data containing crystal data tables, hydrogen bonds, thermal analysis, FTIR spectroscopy and PXRD results can be accessed online at: https://www.mdpi.com/article/10.3390/molecules28196965/s1. The crystallographic data have been deposited with the CCDC, deposition numbers 2286796-2286801. These data can be obtained free of charge from the Cambridge Crystallographic Data Centre via www.ccdc.cam.ac.uk/data_request/cif.

## Supplementary Materials

The following supporting information can be downloaded at: https://www.mdpi.com/article/10.3390/molecules28196965/s1, Figure S1: TG and DSC curves for (a) (CHPAA^2−^)(2DEA^+^) and (b) (CHPAA^−^)(DBM^+^); DSC curves for (c) (CHPAA^−^)(A2MP^+^), (d) (CHPAA^−^)(A24MP^+^), (e) (2CHPAA^−^)(2A26MP^+^) and (f) (CHPAA^−^)(DMAP^+^); Figure S2: FTIR spectroscopy of (a) CHPAA, (b) (CHPAA^2−^)(2DEA^+^) and (c) (CHPAA^−^)(2DBM^+^); Figure S3: FTIR spectroscopy of (a) CHPAA, (b) A2MP, (c) (CHPAA^−^)(A2MP^+^), (d) A24MP, (e) (CHPAA^−^)(A24MP^+^), (f) A26MP, (g) (2CHPAA^−^)(2A26MP^+^)CHPAA), (h) DMAP and (i) (CHPAA^−^)(DMAP^+^); Figure S4: PXRD analyses for (a) (CHPAA^2−^)(2DEA^+^), (b) (CHPAA^−^)(DBM^+^), (c) (CHPAA^−^)(A2MP^+^), (d) (CHPAA^−^)(A24MP^+^), (e) (2CHPAA^−^)(2A26MP^+^) and (f) (CHPAA^−^)(DMAP^+^); Table S1: Summary of crystallographic data; Table S2: Summary of hydrogen bonds; Table S3: Summary of the various interactions; Table S4: DSC and TGA results of CHPAA linear salts; Table S5: DSC results of CHPAA aromatic salts and starting materials.

## Sample Availability

Samples of the compounds are not available from the authors.
