# Supplementary material for: Crystal Structures and Physicochemical Properties of 3-Chloro-4-hydroxyphenylacetic Acid Salts with Amines
Source: Molecules. 2023 Oct 7;28(19):6965. doi: 10.3390/molecules28196965 (PMC10574338; doi:10.3390/molecules28196965)
Supplement: Supplementary file 1 [file molecules-28-06965-s001.zip › molecules-2598014-supplementary.pdf]

Crystal structures and physicochemical properties of 3-chloro-4-hydroxyphenylacetic acid salts with amines

SUPPLEMENTARY DATA

Remi Rolland Ngoma Tchibouanga[a] *and Ayesha Jacobs*[a,*]

[a]Chemistry Department, Faculty of Applied Sciences, Cape Peninsula University of Technology, PO Box 1906, Bellville 7535, South Africa

*E-mail: jacobsa@cput.ac.za

INDEX

**1.1S Crystal Structure Data**	**page 3**
**1.2S Hydrogen Bonding Table**	**page 4**
**1.3S Hirshfeld Surface Analysis**	**page 6**
**1.4S Thermal analysis**	**page 7**
**1.5S FTIR spectroscopy**	**page 9**
**1.6S Powder X-ray diffraction**	**page 10**

1.1S: Crystal Structure Data

Table S1 Summary of crystallographic data

**Salts**	**1**	**2**	**3**	**4**	**5**	**6**
Code	**($CHPAA^{2-}$)(2DEA^+)**	**($CHPAA^-$)(DBM^+)**	**($CHPAA^-$)($A2MP^+$)**	**($CHPAA^-$)($A24MP^+$)**	**(2$CHPAA^-$)(2$A26MP^+$)**	**($CHPAA^-$)($DMAP^+$)**
Structural formula	$C_{16}H_{29}ClN_2O_3$	$C_{16}H_{26}ClNO_3$	$C_{13}H_{13}ClN_2O_3$	$C_{14}H_{15}ClN_2O_3$	$C_{14}H_{15}ClN_2O_3$	$C_{15}H_{17}ClN_2O_3$
Molecular mass (g mol^{-1})	332.86	315.83	280.70	294.74	294.73	308.76
Data collection temp. (K)	173(2)	173(2)	173(2)	173(2)	173(2)	173(2)
Crystal size (mm)	0.30x0.20x0.10	0.21x0.16x0.12	0.24x0.20x0.19	0.32x0.22x0.15	0.22x0.13x0.12	0.32x0.24x0.10
Space group	$P\bar{1}$	C2/*c*	$P\bar{1}$	$P2_1/c$	$P\bar{1}$	*Pbca*
a (Å)	8.0728(16)	22.063(4)	8.9470(18)	8.9433 (18)	8.0965(16)	10.042(2)
b (Å)	8.9900(18)	13.192(3)	9.0283(18)	12.556(3)	8.7236(17)	14.734(3)
c (Å)	13.506(3)	14.638(3)	9.0599(18)	12.940(3)	20.743(4)	19.827(4)
α (°)	87.09	90.00	78.52(3)	90.00	87.61(3)	90.00
β (°)	81.25	126.49(3)	76.89(3)	101.52(3)	88.18(3)	90.00
γ (°)	70.27(3)	90.00	67.78(3)	90.00	68.67(3)	90.00
Volume ($Å^3$)	911.9(3)	3425.4(12)	654.6(2)	1423.7(5)	1363.30(5)	2933.5(10)
Z	2	8	2	4	4	8
D_c, calc density (g cm^{-3})	1.212	1.225	1.424	1 .375	1.436	1.398
Absorption coefficient (mm^{-1})	0.223	0.232	0.297	0.277	0.289	0.272
θ range	1.53-28.32	1.924-27.95	2.33-27.94	2.283-28.306	1.966-28.366	2.054-27.507
Reflections collected	26693	34213	10460	25351	23011	60990
No data I >2 sigma (*I*)	3594	377	2703	2807	4951	2302
Final *R* indices [I >2 sigma (*I*)]	R_1=0.0363; wR_2=0.0961	R_1=0.0344; wR_2=0.0861	R_1=0.0411; wR_2=0.1143	R_1=0.0407; wR_2=0.1016	R_1=0.0708; wR_2=0.1750	R_1=0.0574; wR_2= 0.1647
R indices (all data)	R_1=0.0494; wR_2=0.0887	R_1=0.0453; wR_2=0.0934	R_1=0.0471; wR_2=0.1087	R_1=0.0537; wR_2=0.1098	R_1=0.0971; wR_2=0.1867	R_1=0.0973; wR_2= 0.2303
Goodness-of-fit on F^2	1.030	1.026	1.043	1.068	1.089	1.057
CCDC no.	2286796	2286797	2286798	2286799	2286800	2286801

1.2S: Hydrogen bonding

Table S2 Summary of hydrogen bonds

	**D-H (Å)**	**H⋯A (Å)**	**D⋯A (Å)**	**<DHA (º)**	**Symmetry operations**
$(CHPAA^{2-})(2DEA^{+})$					
N1-H1⋯O1	0.929(16)	1.826(16)	2.7171(15)	159.9(14)	
N2-H1A⋯O3	0.873(19)	1.966(19)	2.7573(16)	150.1(16)	
N1-H2⋯O1	0.984(17)	1.678(18)	2.6520(17)	169.4(15)	1-x, 1-y, -z
N2-H2A⋯O3	1.019(18)	1.666(19)	2.6834(17)	176.3(17)	1-x, -y, 1-z
C3-H3⋯O2	0.95	2.73	3.613(2)	155.1	1-x, 1-y, 1-z
C11-H112⋯O2	0.99	2.54	3.4706(18)	157.3	x, y, z-1
C11-H111⋯Cl1	0.99	2.98	3.821(2)	143.3	2-x, -y, -z
C11A-H11A⋯Cl1	0.99	2.95	3.913(2)	165.8	
N1-H1⋯Cl1	0.929(16)	2.857(15)	3.4818(16)	125.7(11)	
$(CHPAA^{-})(DBM^{+})$					
N1-H17⋯O3	0.935(15)	1.828(16)	2.7526(14)	169.4(14)	
N1-H18⋯O3	0.933(17)	1.836(17)	2.7419(19)	163.0(14)	-x, y, ½-z
O1-H1⋯O2	0.86(2)	1.75(2)	2.6069(16)	173.4(19)	½+x, -y-½, ½+z
C10-H10A⋯Cl1	0.99	3.09	3.7662(15)	126.5	-x, y, ½-z
C16-H16C⋯Cl1	0.98	3.12	3.6809(17)	117.6	-x, y-1, ½-z
$(CHPAA^{-})(A2MP^{+})$					
N2-H14⋯O2	0.81(2)	2.08(2)	2.870(2)	163(2)	
N2-H15⋯O2	0.84(2)	2.03(2)	2.8556(19)	167.4(18)	1-x, 2-y, 2-z
N1-H16⋯O3	0.90(2)	1.83(2)	2.7294(17)	178.7(17)	1-x, 2-y, 2-z
O1-H1⋯O3	0.81(2)	1.86(2)	2.6530(19)	170(2)	x-1, y, z
C10-H10⋯O1	0.95	2.50	3.367(2)	151.6	-x, 1-y, 2-z
C9-H9⋯O3	0.95	2.53	3.461(2)	164.8	x-1, y, 1+z
C10 H10⋯Cl1	0.95	3.06	3.7854(17)	134.4	-x, 1-y, 2-z
$(CHPAA^{-})(A24MP^{+})$					
N1-H15⋯O3	0.88(2)	1.85(2)	2.7167(18)	168.9(18)	
N2-H13A⋯O3	0.89(2)	2.01(2)	2.8875(19)	169(2)	x, 3/2-y, z-½
O1-H1⋯O2	0.91(3)	1.71(3)	2.6134(17)	173(3)	1-x, 2-y, -z
C14-H14⋯O1	0.938(19)	2.506(19)	3.405(2)	160.5(15)	1+x, 3/2-y, z-½
C7-H7A⋯O2	0.99	2.61	3.527(2)	154.7	2-x, 2-y, -z
C3-H3⋯Cl1	0.95	3.22	3.5088(16)	99.9	1+x, ½+y, ½-z

	**D-H (Å)**	**H⋯A (Å)**	**D⋯A (Å)**	**<DHA (º)**	**Symmetry operations**
$(2CHPAA^{-})(2A26MP^{+})$					
N2-H2B⋯O2A	0.85(4)	2.03(4)	2.856(5)	163(4)	2-x, 1-y, 1-z
N2-H2A⋯O2A	0.97(6)	1.84(6)	2.783(4)	163(5)	x-1, y, z
N1-H68⋯O3A	0.87(5)	1.96(5)	2.821(4)	172(4)	x-1, y, z
N2A-H2A'⋯O2	0.90(5)	1.92(5)	2.798(4)	166(4)	x-1, y, z
N2A-H2B'⋯O2	0.91(5)	1.97(5)	2.863(4)	166(4)	1-x, 2-y, -z
N1A-H67⋯O3	0.90(4)	1.93(4)	2.812(4)	166(3)	x-1, y, z
O1-H2⋯O3	0.91(5)	1.77(5)	2.678(4)	178(4)	x, y-1, z
O1A-H2'⋯O3A	0.85(5)	1.87(5)	2.677(4)	159(5)	x, y-1, z
C9-H9A⋯O1A	0.98	2.56	3.523(5)	166.1	x, 1+y, z
C9A-H9A'⋯O1	0.98	2.56	3.507(4)	163.5	x, 1+y, z
C9A-H9A'⋯Cl1	0.98	3.10	3.823(4)	131.5	x, 1+y, z
$(CHPAA^{-})(DMAP^{+})$					
N1-H1A⋯O3	0.88	1.80	2.665(3)	168.2	$^{3}/_{2}$-x, y-½, z
O1-H1⋯O2	0.84	1.73	2.568(3)	171.2	1-x, y-½, $^{3}/_{2}$-z
C10-H10⋯O1	0.95	2.53	3.281(3)	136.1	
C12-H12⋯O3	0.95	2.59	3.385(3)	141.6	1-x, -y, 1-z
C7-H7B⋯O1	0.99	2.50	3.386(3)	148.3	½ -x, ½+y, z
C15-H15A⋯O3	0.98	2.40	3.371(4)	168.6	1-x, -y, 1-z
C4-H4⋯Cl1	0.95	2.93	3.595(3)	128.3	½+x, y, $^{3}/_{2}$-z
C13-H13⋯Cl1	0.95	2.86	3.473(3)	123.3	1-x, -y, 1-z
C14-H14A⋯Cl1	0.98	2.85	3.756(3)	154.3	-x, -y, 1-z
C15-H15B⋯Cl1	0.98	2.89	3.846(4)	164.4	-x, -y, 1-z

1.3S Hirshfeld Surface Analysis

Table S3 Summary of the various interactions

Compound	O···H	H···H	C···H	Cl···H	Cl···Cl	C···C
$(CHPAA^{2-})(2DEA^{+})$	33.9%	33%	16.3%	16%	0.8%	-
$(CHPAA^{-})(DBM^{+})$	32.9%	32.9%	15.3%	18.8%	-	-
$(CHPAA^{-})(A2MP^{+})$	33%	25.2%	16.2%	15.8%	-	4.8%
$(CHPAA^{-})(A24MP^{+})$	31.1%	29%	14.1%	16.8%	-	3.2%
$(2CHPAA^{-})(2A26MP^{+})$	31.1%	27.2%	19%	16.4%	-	-
	31.9%	25.6%	20%	16.7%	-	-
$(CHPAA^{-})(DMAP^{+})$	33.5%	29.2%	18.1%	15.9%	-	-

1.4S Thermal analysis

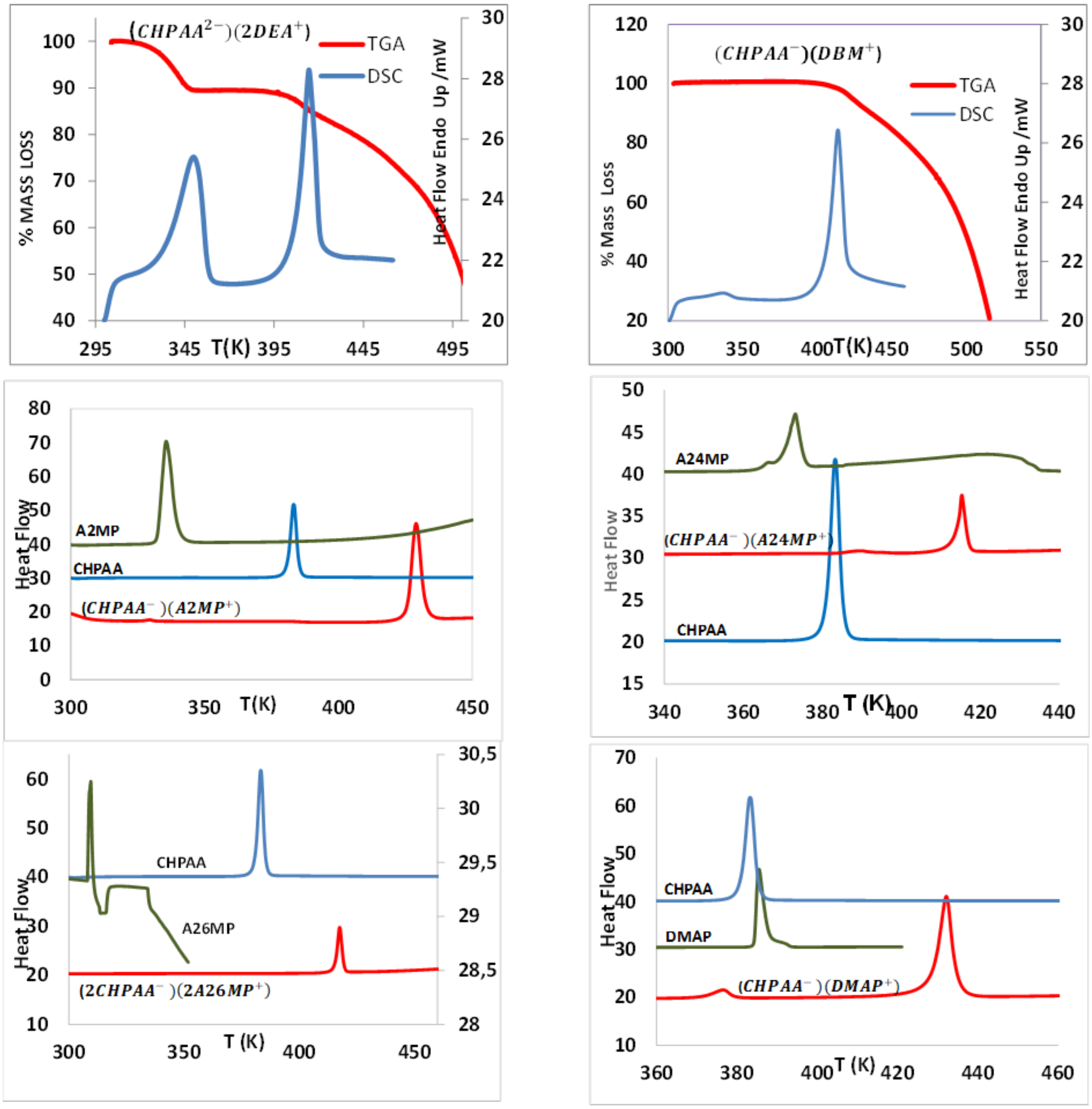

Figure S1: TG and DSC curves for (a) $(CHPAA^{2-})(2DEA^{+})$ and (b) $(CHPAA^{-})(DBM^{+})$; DSC curves for (c) $(CHPAA^{-})(A2MP^{+})$, (d) $(CHPAA^{-})(A24MP^{+})$, (e) $(2CHPAA^{-})(2A26MP^{+})$ and (f) $(CHPAA^{-})(DMAP^{+})$.

Table S4 DSC and TGA results of CHPAA linear salts

Compound	$(CHPAA^{2-})(2DEA^{+})$	$(CHPAA^{-})(DBM^{+})$
Host:Guest ratio	1:2	1:1
TG theo % mass loss	44.4	41
Exp % mass loss	10.5 (Initial step)	/
DSC endotherm for loss of solvent (T_{On}/K)	316	/
DSC endotherm for the melt of CHPAA (T_{on}/K)	391	392(including loss of remaining DBM)
Solvent normal bp K	329	433

Table S5 DSC results of CHPAA aromatic salts and starting materials

Compound	DSC endotherm 1(K)	DSC endotherm 2 (K)
(CHPAA)	378.3	/
$(CHPAA^{-})(A2MP^{+})$	423.3	/
$(CHPAA^{-})(A24MP^{+})$	410.7	/
$(2CHPAA^{-})(2A26MP^{+})$	414.8	/
$(CHPAA^{-})(DMAP^{+})$	373.2 (melt of residual CHPAA)	427.6

1.5S FTIR spectroscopy

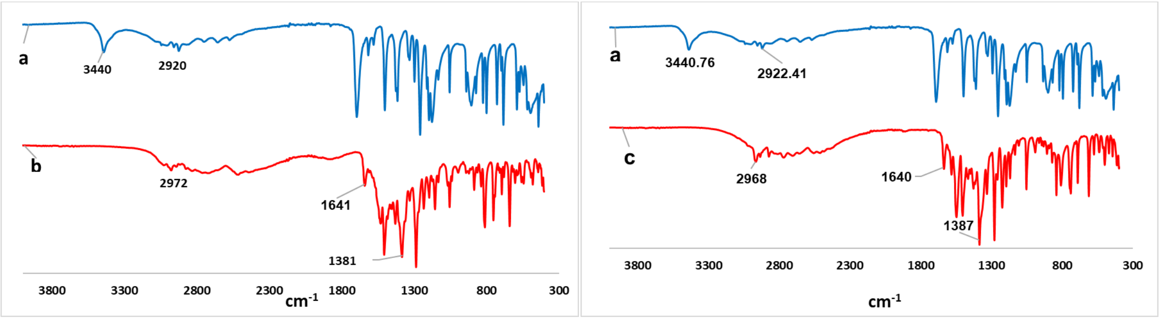

Figure S2: FTIR spectroscopy of (a) CHPAA, (b) ($CHPAA^{2-}$)($2DEA^{+}$) and (c) ($CHPAA^{-}$)($2DBM^{+}$)

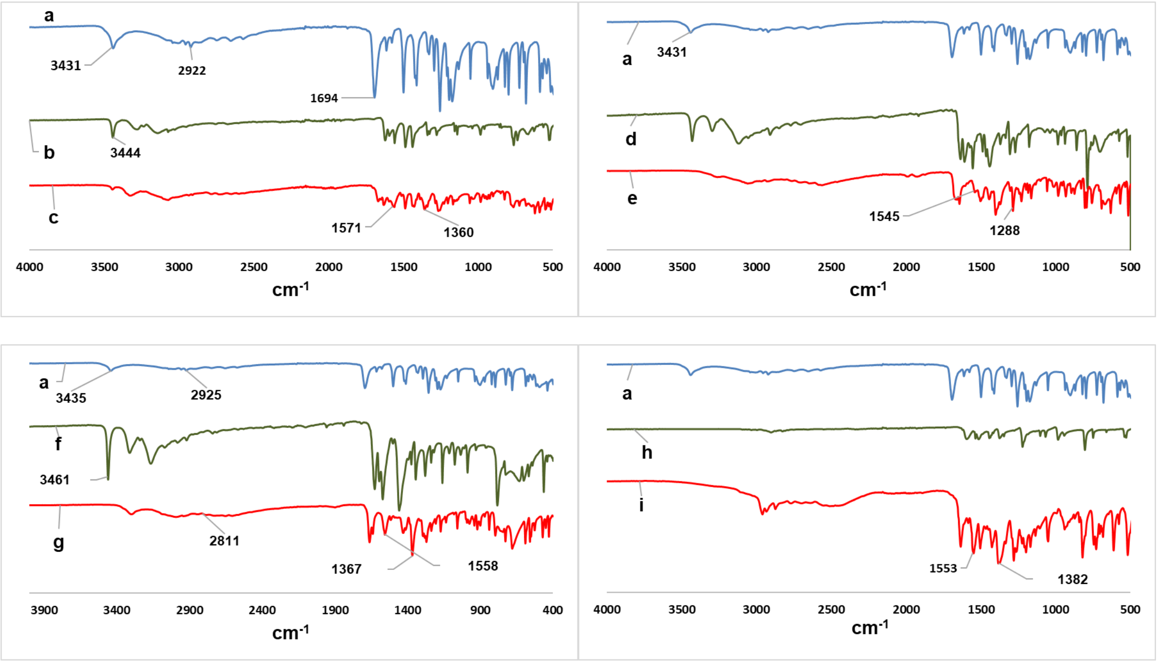

Figure S3: FTIR spectroscopy of (a) CHPAA, (b) A2MP, (c) ($CHPAA^{-}$)($A2MP^{+}$), (d) A24MP, (e) ($CHPAA^{-}$)($A24MP^{+}$), (f) A26MP, (g) ($2CHPAA^{-}$)($2A26MP^{+}$)CHPAA), (h) DMAP and (i) ($CHPAA^{-}$)($DMAP^{+}$)

1.6S Powder X-ray diffraction

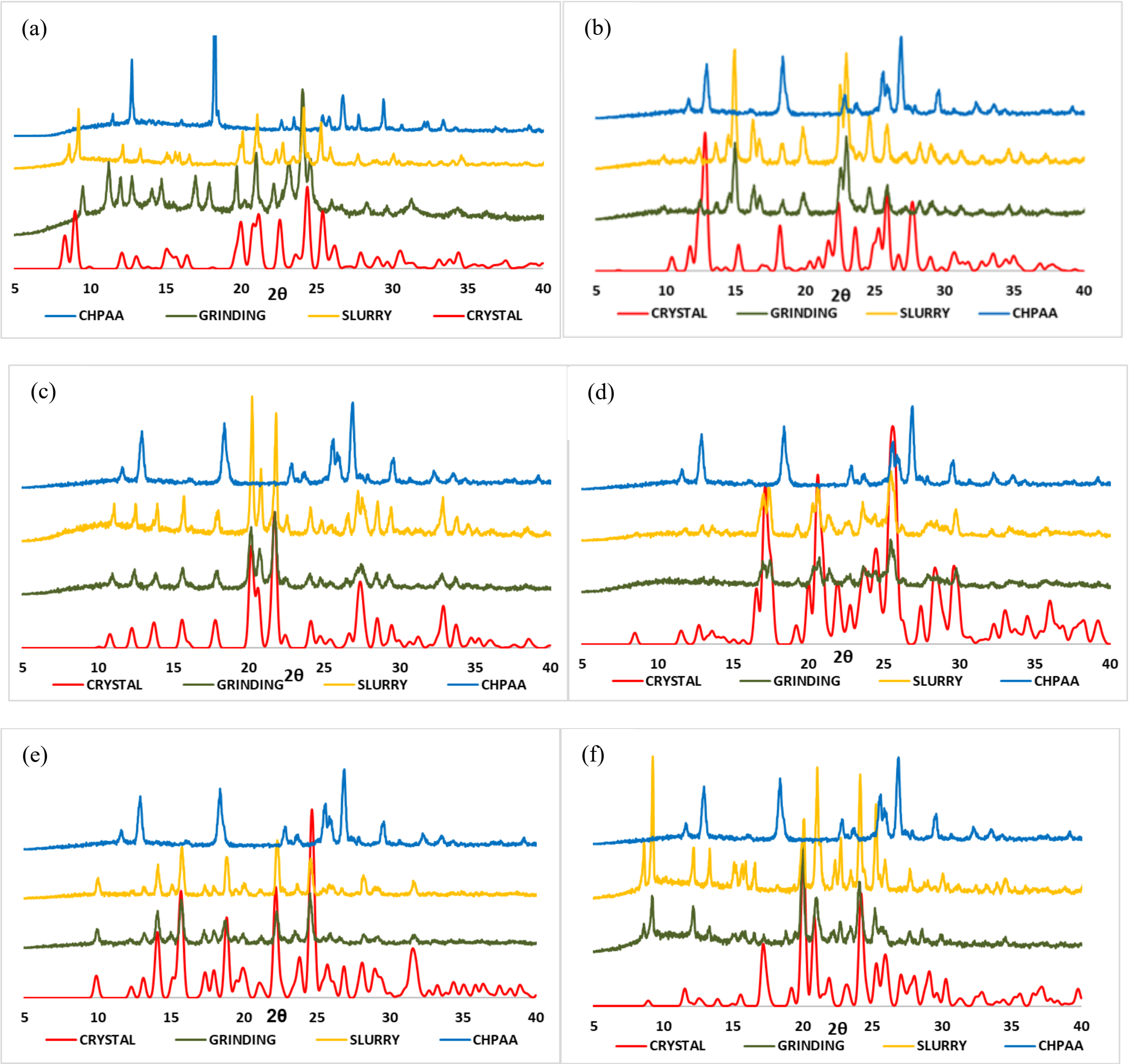

Figure S4: PXRD analyses for (a) (CHPAA^{2-})(2DEA^{+}), (b) (CHPAA^{-})(DBM $^{+}$), (c) (CHPAA^{-})(A2MP^{+}), (d) (CHPAA^{-})(A24MP^{+}), (e) (2CHPAA^{-})(2A26MP^{+}) and (f) (CHPAA^{-})(DMAP^{+}).
